# Supplementary figures and images for: Neutralizing antibodies to Omicron after the fourth SARS-CoV-2 mRNA vaccine dose in immunocompromised patients highlight the need of additional boosters
Source: Front Immunol. 2023 Jan 27;14:1104124. doi: 10.3389/fimmu.2023.1104124 (PMC9911671; doi:10.3389/fimmu.2023.1104124)

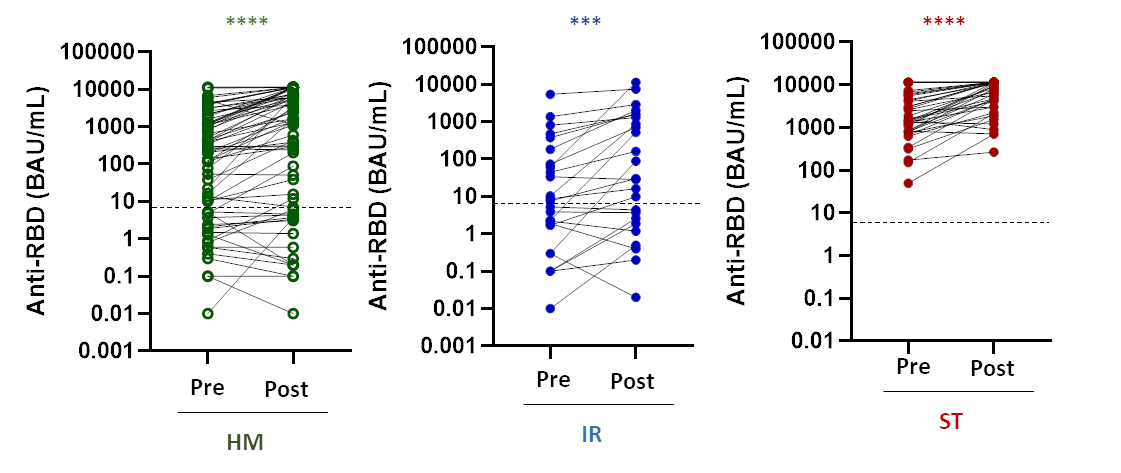

Supplement: Supplementary Figure 1 — SARS-CoV-2 specific anti-RBD Abs per individual patient. Anti-RBD were measured in sera samples of HM (green dots), IR (blue dots) and ST (red dots) patients before (pre) and after (post) the fourth dose of mRNA vaccine. The level of anti-RBD Abs was expressed as BAU/mL. Differences between anti-RBD titre before and after vaccination were evaluated by Wilcoxon paired test. ****P<0.0001, *** P<0.001. HM, hematological malignancies; IR, immune-rheumatological diseases; ST, solid tumors. [file Image_1.tiff]

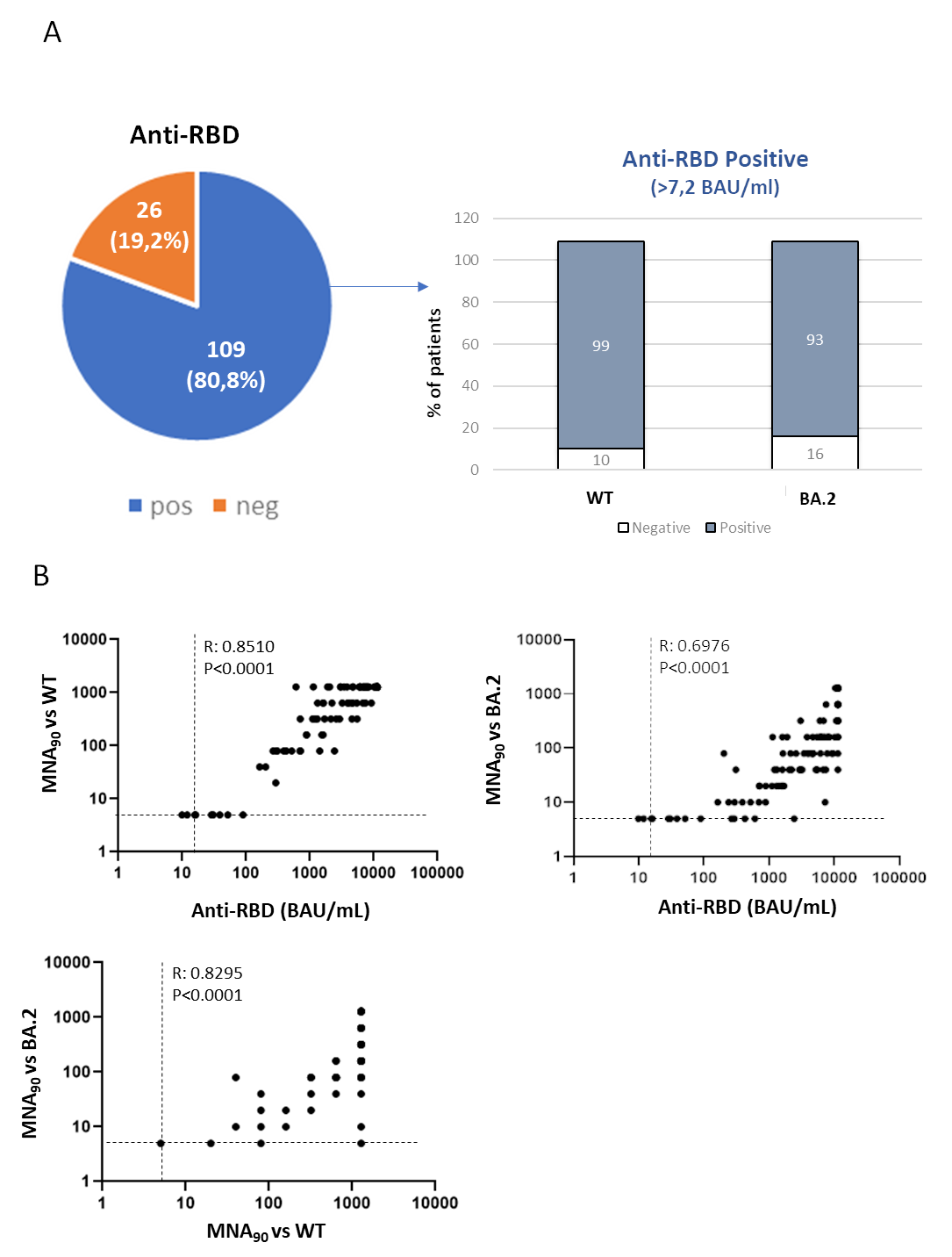

Supplement: Supplementary Figure 2 — Cross reactivity of anti-RBD Abs induced by vaccination. (A) The number (percentage) of patients showing a negative or positive anti-RBD response (cut-off 7.2 BAU/mL) after the fourth dose of mRNA vaccine is shown. Patients with a positive anti-RBD response were further divided in MNA negative (white bars) and positive (grey bars) on the basis of their neutralization capability against WT and BA.2 viral strains (cut-off 5 MNA90). Results are shown as the percentage of MNA90 negative and positive patients and the absolute number of patients are shown within the bars. (B) The correlation between the levels of anti-RBD Abs and neutralization titre (WT or BA.2) after the fourth dose as well as the correlation between the neutralization titre against WT and BA.2 viral strains are shown. Each black dot represents one sample. The analysis was performed by using the Spearman test and Rho and p values are indicated in the figure. [file Image_2.tiff]

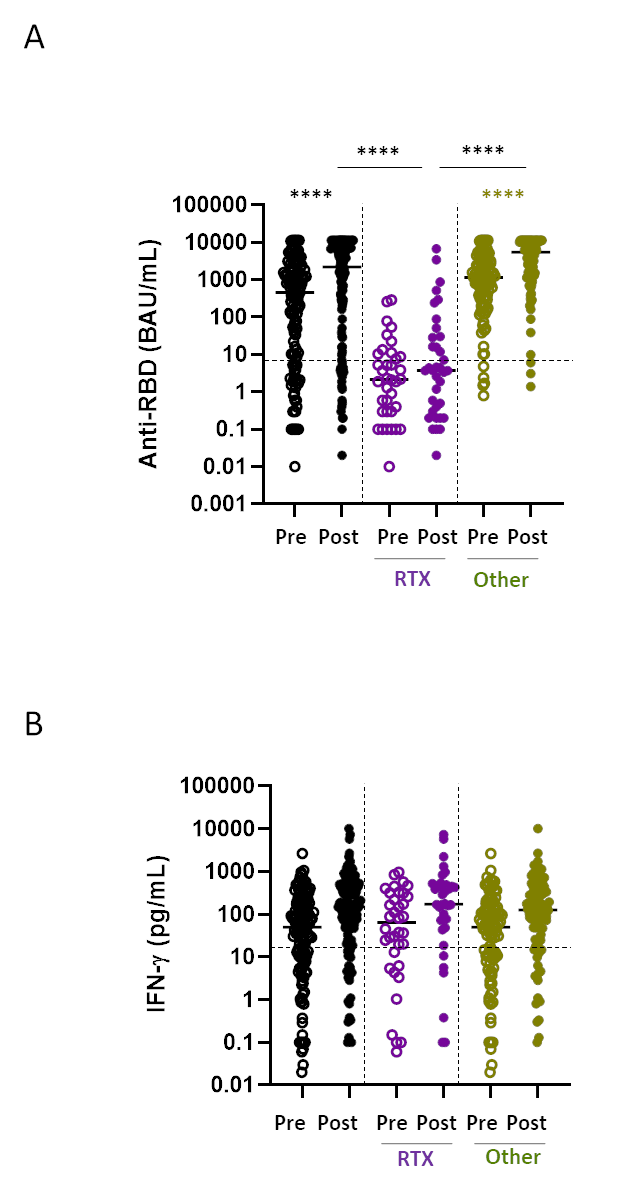

Supplement: Supplementary Figure 3 — Impact of Rituximab therapy on humoral and T cell response (A) SARS-CoV-2 specific anti-RBD Abs before (pre) and after (post) the fourth dose of vaccine were compared in all enrolled patients receiving Rituximab (RTX) or other treatments. All-pre median= 462.5 BAU/mL (IQR: 10.4-1913 BAU/mL); all-post median= 2212 BAU/mL (IQR: 51.6-8391 BAU/mL). RTX-pre median= 2.1 BAU/mL (IQR: 0.3-10.0 BAU/mL); RTX-post median= 3.7 BAU/mL (IQR: 0.3-30.1). Other-pre median=1155 BAU/mL (IQR: 316.4-3145 BAU/mL); Other-post median=5446 BAU/mL (IQR: 1537-11360 BAU/mL) (B) SARS-CoV-2 specific T cell response before (pre) and after (post) the fourth dose of vaccine were compared in all enrolled patients receiving Rituximab (RTX) or other treatments. All-pre median= 49.5.0 pg/mL (IQR: 8.96-177.1 pg/mL); all-post median= 147.0 pg/mL (IQR: 46.7-439.1 pg/mL). RTX-pre median= 63.0 pg/mL (IQR: 7.9-298.7 pg/mL); RTX-post median= 171 pg/mL (IQR: 53.9-475.0 pg/mL). Other-pre median= 49.0 pg/mL (IQR: 8.9-148 pg/mL); Other-post median= 124.5 pg/mL (IQR: 32.6-345.0 pg/mL). [file Image_3.tiff]
